# Comparative Analysis of Cystatin Superfamily in Platyhelminths

**DOI:** 10.1371/journal.pone.0124683

**Published:** 2015-04-08

**Authors:** Aijiang Guo

**Affiliations:** 1 State Key Laboratory of Veterinary Etiological Biology, Key Laboratory of Veterinary Parasitology of Gansu Province, Lanzhou Veterinary Research Institute, Chinese Academy of Agricultural Sciences, Lanzhou, Gansu, China; 2 Jiangsu Co-innovation Center for Prevention and Control of Important Animal Infectious Disease, Yangzhou, Jiangsu, China; University of Minnesota, UNITED STATES

## Abstract

The cystatin superfamily is comprised of cysteine proteinase inhibitors and encompasses at least 3 subfamilies: stefins, cystatins and kininogens. In this study, the platyhelminth cystatin superfamily was identified and grouped into stefin and cystatin subfamilies. The conserved domain of stefins (G, QxVxG) was observed in all members of platyhelminth stefins. The three characteristics of cystatins, the cystatin-like domain (G, QxVxG, PW), a signal peptide, and one or two conserved disulfide bonds, were observed in platyhelminths, with the exception of cestodes, which lacked the conserved disulfide bond. However, it is noteworthy that cestode cystatins had two tandem repeated domains, although the second tandem repeated domain did not contain a cystatin-like domain, which has not been previously reported. Tertiary structure analysis of *Taenia solium* cystatin, one of the cestode cystatins, demonstrated that the N-terminus of *T*. *solium* cystatin formed a five turn α-helix, a five stranded β-pleated sheet and a hydrophobic edge, similar to the structure of chicken cystatin. Although no conserved disulfide bond was found in *T*. *solium* cystatin, the models of *T*. *solium* cystatin and chicken cystatin corresponded at the site of the first disulfide bridge of the chicken cystatin. However, the two models were not similar regarding the location of the second disulfide bridge of chicken cystatin. These results showed that *T*. *solium* cystatin and chicken cystatin had similarities and differences, suggesting that the biochemistry of *T*. *solium* cystatin could be similar to chicken cystatin in its inhibitory function and that it may have further functional roles. The same results were obtained for other cestode cystatins. Phylogenetic analysis showed that cestode cystatins constituted an independent clade and implied that cestode cystatins should be considered to have formed a new clade during evolution.

## Introduction

Chicken cystatin was first described by Barrett, who discovered that it inhibited various cysteine proteases. Subsequently, many similar molecules present in metazoans and plants have been identified [1–3]. The cystatin superfamily includes inhibitors of diverse C1 and C13 families of cysteine proteases [4]. These peptidases play key roles in physiological processes [5–8]. The cystatin superfamily can be assigned to three distinct protein families based on similarities in their amino acid sequences and 3D structures [9]. In this system, the cystatin superfamily is placed in the I25 family, which contains three subfamilies: I25A (type 1, stefins), I25B (type 2, cystatins) and I25C (type 3, kininogens) [10]. Stefins are unglycosylated proteins with an approximate molecular weight of 11 kDa and have no signal sequence or disulfide bonds. They are predominately intracellular and are involved in the regulation of endogenous proteins in cells. The cystatins are about 13–14 kDa in size and have a signal sequence and two carboxy-terminal disulfide bonds. They are usually exported from cells and participate in regulation of the exogenous proteins [4]. The kininogens are restricted to the vertebrates [11]. The cystatin superfamily features numerous important common characteristics, but the differences in molecular structure and distribution imply different routes of biosynthesis and a variety of physiological functions.

Recently, some studies have described the capacity of nematode cystatins to regulate not only the activity of parasite proteases but also that of host proteases [12]. Additionally, some studies have shown that nematode cystatins modulate host immune responses [13–16]. However, little is known about the cestode cystatin superfamily and trematode cystatin superfamily [17–19].

Cysteine proteases have been described in cestodes and trematodes [20–23]. The expression of cysteine proteases implies the presence of members of the cystatin family [5]. Early attempts to infer molecular phylogenies of the cystatin superfamily have been hampered by the unavailability of genomic sequences. With the rapid development of next-generation sequencing technology, and the availability of complete genome and transcriptome data from an increasing number of parasite species, it is now feasible to explore cystatin superfamily genomic organization, classification and phylogeny.

In the present study, the cystatin superfamily was characterized and explored for their phylogeny in nine flatworms including *Schmidtea mediterranea*, *Schistosoma japonicum*, *Schistosoma mansoni*, *Echinococcus multilocularis*, *Echinococcus granulosus*, *Hymenolepis microstoma*, *T*. *solium*, *T*. *saginata* and *T*. *asiatica*. With the advantage of having *T*. *solium*, *T*. *saginata* and *T*. *asiatica* genomic data and samples, their corresponding cystatin superfamily was cloned and analyzed, which may provide a comprehensive insight into its abundance, diversity and evolution in platyhelminths.

## Materials and Methods

### Annotated genomes of platyhelminth species

The annotated genomes of 6 platyhelminths are available for public study. Those of the trematodes *S*. *japonicum* and *S*. *mansoni* and the cestodes *T*. *solium*, *E*. *multilocularis*, *E*. *granulosus* and *H*. *microstoma* can be found at http://www.genedb.org/homepage. The genome of the planarian *S*. *mediterranea* is available at http://smedgd.neuro.utah.edu. Each genome was investigated using the word “cystatin” as the query parameter. The expressed sequence tags (ESTs) or RNA-Seq data of each species were searched for the details of their transcription. For full-scale analysis, the nr and EST databases of NCBI (http://blast.ncbi.nlm.nih.gov/) were also included in the searches.

### Identification of cystatin superfamily in *T*. *solium*, *T*. *saginata* and *T*. *asiatica*

The unpublished genome sequences of *T*. *saginata* and *T*. *asiatica* were used for the identification of the cystatin family. A combined strategy was employed using both the motifs-based method and the sequence similarity-based method to search for proteins or molecules with conserved cystatin-like domains in the genomes of the two tapeworms. Putative members of the cystatin superfamily in *T*. *saginata* and *T*. *asiatica* were characterized using the TBlastN program, with the annotated *T*. *solium* cystatin superfamily gene sequences (TsM_000671000 and TsM_000687900) as query sequences, to search the two genome databases with the cut-off e-value of 10^−5^ [24]. In addition, corresponding amino acid sequences were analyzed by the MEROPS peptidase database (http://merops.sanger.ac.uk/) and those belonging to proteinase inhibitor I25 [25] were considered for the putative cystatin superfamily.

The DNA and cDNA samples of *T*. *solium*, *T*. *saginata* and *T*. *asiatica* are available in our lab [24, 26]. In order to investigate gene organizations of the cystatin superfamily in these three species, their corresponding putative cystatin superfamily genes were amplified using DNA or cDNA as templates, under the thermal cycling profile of 94°C for 5 min, 30 cycles of denaturation at 94°C for 40s, annealing at 55°C for 30s and extension at 72°C for 50s. The amplified fragments were cloned into pGEM-T Easy Vector (Promega, USA) and confirmed by DNA sequencing (Takara, China). The specific primers are shown in Table 1.

**Table 1:** Primers used for amplifying cystatin superfamily of *T*. *solium*, *T*. *saginata* and *T*. *asiatica*.

Name	Forward primer	Downward primer
*T*. *solium*, *T*. *saginata* and *T*. *asiatica* cystatin	5’- ATGAATTGGTCTGTTCTTCTGCTACTC-3’	5’- TCATAGGGTAGCTGGGCCTTTG-3’
*T*. *solium* stefin	5’-ATGCCGATGTGTGGTGGTTTG-3’	5’-TTAAAAGTATGTCAGAGGGTCTCCAG-3’
*T*. *saginata* stefin	5’-ATGCCCAGGTGTGGTGGTTTG-3’	5’-TTAGAAGTAAGTCAACGGATCTCCAG-3’
*T*. *asiatica* stefin1	5’-ATGCCGATGTGTGGTGGCTTG-3’	5'-TTAAAAGTATGTCAGTGGGTCTCCAG-3'
*T*. *asiatica* stefin2	5'-ATGCAGGATTCGCGAGCGATTA-3'	5'-TTAAAAGTAAGTCAACGGATCTCCAGC-3'

### Analysis of amino acid sequences and 3D models of platyhelminth cystatin superfamily

Signal peptides were predicted using SignalP 4.0 (www.cbs.dtu.dk/services/SignalP/) [27]. Disulfide bonds and domain architecture were predicted using the DiANNA 1.1 web server [28, 29] and SMART (http://smart.embl-heidelberg.de) [30], respectively. The predicted 3D models of platyhelminth stefins and cystatins were constructed by the homology-modeling server SWISS-MODEL (http://swissmodel.expasy.org/) [31]. The templates for the models were auto-selected by servers (chicken cystatin, PDB ID: 1yvb.1.B; others using 4lzi.1.A as model) [32–36]. VMD was used to display the tertiary structures of cystatins.

### Sequence alignment and phylogenetic analysis

Protein sequences were aligned using ClustalW2 [37] and manually checked. The optimal model of protein evolution was selected by ProtTest [38]. The phylogenetic tree of flatworm cystatin superfamily members was constructed by the Maximum Likelihood (ML) method of PhyML [39] and with the following conditions: JII+I+G and the reliability of each branch was assessed by performing 100 bootstrap replications.

## Results and Discussion

### Identification of cystatin superfamily in platyhelminths

**Fig 1:**
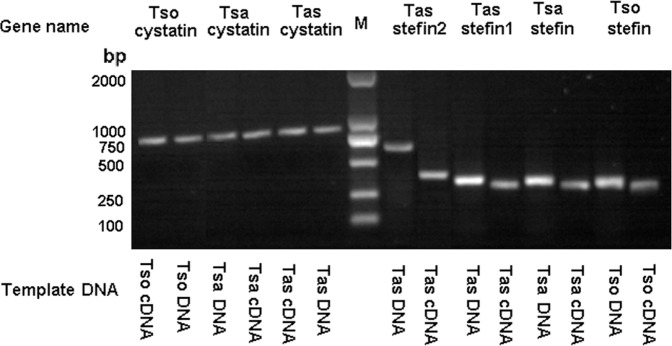
PCR fragments for cystatin superfamily in *T*. *solium*, *T*. *saginata* and *T*. *asiatica*. DNA and cDNAs of the three species were used as templates to amplify cystatin superfamily. The PCR products of cystatins are shown on the left of the DL2000 molecular marker, and stefins are shown on the right.

**Fig 2:**
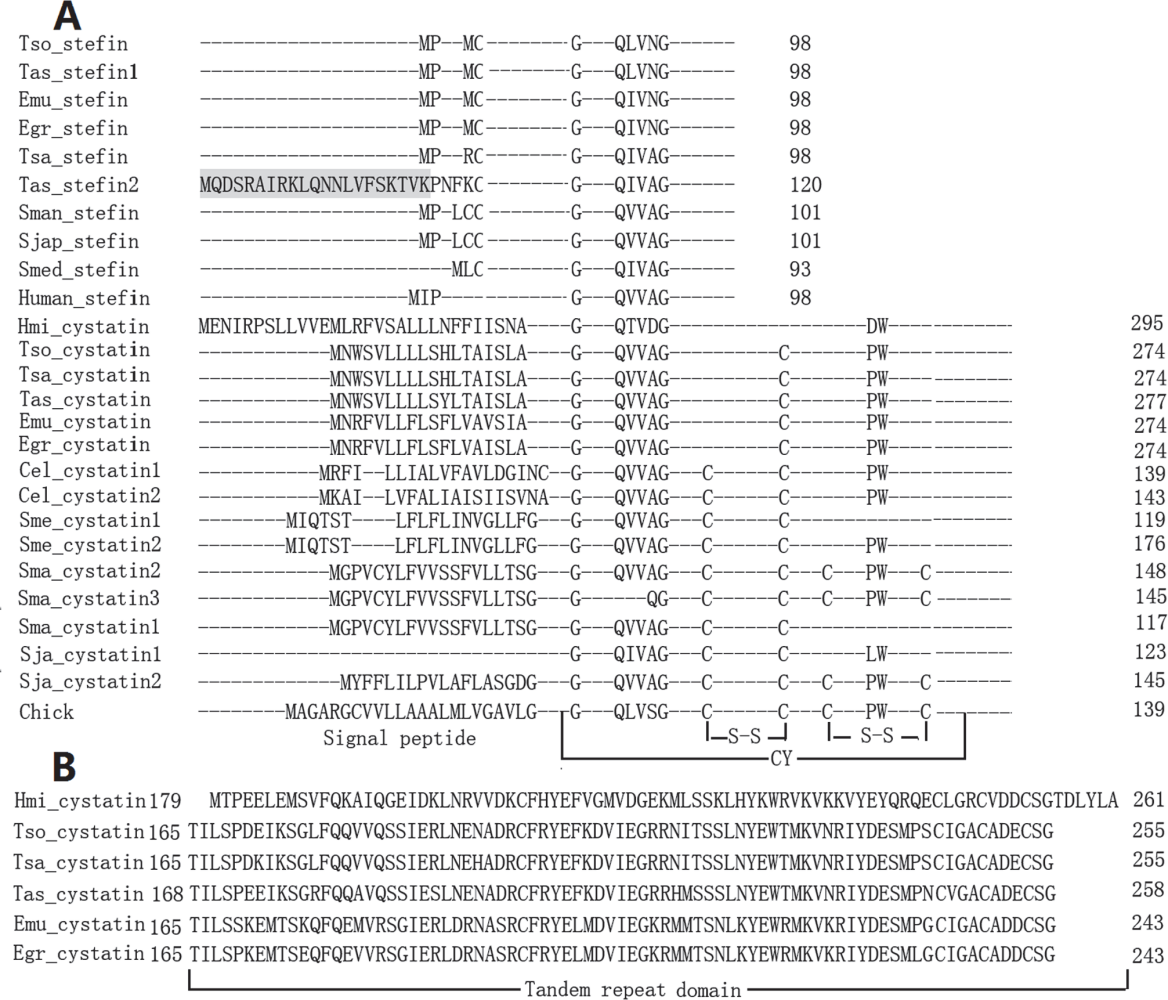
Alignment of cystatin superfamily domain. The domain was aligned using ClustalW and adjusted manually. (A) The three parts of the papain binding domain (G, QXVXG, PW) are displayed. Signal peptides and disulfide bridges are shown. The N-terminal amino acid extention of Tas_stefin2 is shaded pale grey. The number of amino acids of each protein is shown at last of sequence. (B) The tandem repeated domain of cestode cystatins is shown. The first amino acid and the last amino acid in the tandem repeated domain is numbered outside of them.

Cystatin superfamily members were identified in platyhelminths and are shown in Table 2. One stefin and one cystatin were characterized in each of *T*. *solium* and *T*. *saginata*, whereas one cystatin and two stefins were found in the *T*. *asiatica* genome. The expression of the stefins and the cystatins in the above mentioned three species was then confirmed by PCR using their corresponding cDNA as template (Fig 1). Similarly, the putative cystatin superfamily members were also identified in other platyhelminth species (Table 2). A single stefin gene was identified in each of *E*. *multilocularis*, *E*. *granulosus*, *S*. *japonicum*, *S*. *mansoni* and *S*. *mediterranea*. In addition, a single cystatin gene was found in each genome of tree cestodes including *E*. *multilocularis*, *E*. *granulosus* and *H*. *microstoma*, while two cystatin genes were found in each of *S*. *japonicum* and *S*. *mediterranea*. Furthermore, three different spliced cystatin proteins were deduced from electronic annotation in *S*. *mansoni* (Smp_034420.1, Smp_034420.2 and Smp_034420.3) (Figs 2A and S2). According to analysis of the transcriptomic and genomic data, two ancestral lineages, stefins and cystatins, were present in numerous platyhelminths. This result was in agreement with studies in some eukaryotic species with two ancestral lineages [40], but in disagreement with the early proposition that stefins, cystatins and kininogens appear almost simultaneously [41].

**Table 2:** Cystatin superfamily genes used for the deduced amino acid sequence alignment or phylogenic study in platyhelminths.

Species/Date origin	Acc. no of sequences	Protein name	SP site (aa) ^a^	Gene structure ^b^	Length (aa) ^c^	EST^d^
*T*. *solium* **/**in this study	KJ941089	Stefin	ND	174+(37)+123	98	Yes
	KJ941088	Cystatin	1–18	825	274	Yes
*T*. *saginata* **/**in this study	KJ941090	Stefin	ND	174+(37)+123	98	Yes
	KJ941091	Cystatn	1–18	825	274	Yes
*T*. *asiatica* **/**in this study	KJ941092	Stefin 1	ND	174+(37)+123	98	Yes
	KJ941093	Stefin 2	ND	75+(248)+165+(37)+123	120	Yes
	KJ941094	Cystatin	1–18	834	277	Yes
*E*. *multilocularis* /Gene DB	EmuJ_000159200	Stefin	ND	174+(37)+123	98	No
	EmuJ_000849600	Cystatin	1–18	825	274	No
*E*. *granulosus* /Gene DB	EgrG_000159200	Stefin	ND	174+(37)+123	98	Yes
	EgrG_000849600	Cystatin	1–18	825	274	No
*H*. *microstoma* /Gene DB	HmN_000842000	Cystatin	1–30	888	295	No
*S*. *japonicum* /Gene DB	Sjp_0066340	Stefin	ND	75+(34)+102+(36)+129	101	Yes
	Sjp_0094540	Cystatin 1	ND	177+(55)+123+(9152)+72	123	No
	Sjp_0005780	Cystatin 2	1–18	209+(38)+74+(43)+56+(34)+99	145	Yes
*S*. *mansoni* /Gene DB	Smp_006390	Stefin	ND	75+(34)+104+(37)+127	101	Yes
	Smp_034420.1	Cystatin 1	1–19	218+(36)+74+(40)+62	117	No
	Smp_034420.2	Cystatin 2	1–19	218+(36)+74+(40)+56+(40)+99	148	No
	Smp_034420.3	Cystatin 3	1–19	217+(45)+66+(40)+56+(40)+99	145	No
*C*. *elegans*/NCBI	NC_003282.7	Cystatin 1	1–19	102+(449)+72+(77)+246	139	Yes
	NC_003283.10	Cystatin 2	1–19	111+(120)+78+(888)+243	143	Yes
*S*. *mediterranea* /Gene DB	v31.000249:67611.67740	Stefin	ND	66+(40)+84+(64)+132	93	Yes
	v31.004385:49482.49541	Cystatin 1	1–19	207+(115)+151	119	Yes
	v31.027397:6615.6712	Cystatin 2	1–19	207+(115)+117+(3949)+114+(47)+90	176	Yes

^a^ND: not detected;

^b^For each gene structure, exons and introns (numbers in brackets) are shown;

^c^Number of amino acid residues;

^d^Putative cystatin or stefin expression was validated by transcriptomic or/and EST data or/and cDNA cloning using Blast; SP: Signal peptide.

Interestingly, analysis of the gene structures of the platyhelminth cystatin superfamily showed that cestode cystatins were intronless (Table 2), which was further confirmed in *T*. *solium*, *T*. *saginata* and *T*. *asiatica* by PCR and sequencing (Fig 1 and Table 2). These results were remarkably different from those of other platyhelminths, which had at least one intron (Table 2). Additionally, the intron position and length in each of the cestode stefins were identical, except for *T*. *asiatica* stefin2 (Table 2). Gene organizations for the three parasite stefins were also confirmed through PCR and sequencing (Fig 1 and Table 2).

### Analysis of amino acid sequences of cystatin superfamily in platyhelminths

Cystatins and stefins have one conserved domain that is important for their inhibition of papain-like cysteine proteases [5]. A signal peptide and two characteristic intra-chain disulfide bonds are present in cystatins, which are secreted proteins with 120 amino acid residues, but are absent in stefins with approximately 100 amino acid residues [5].

Compared with a human stefin, the reference for the type 1 subfamily [33], platyhelminth stefins have similar catalytic sites (G, QXVXG) (Fig 2A, also see S1 Fig for a complete list). The size of platyhelminth stefins ranged from 93 to 98 amino acids that are commonly found in mammalian stefins. The exception was *T*. *asiatica* stefin2, which contains 121 amino acids (Fig 2A). Compared with other platyhelminth stefins, *T*. *asiatica* stefin2 had a 25 amino acid extension at the N-terminus (Fig 2A, also see S1 Fig for a complete list) which was not a signal peptide. Comparisons of amino acid sequences of *T*. *asiatica* stefin1 with *T*. *solium* stefin and *T*. *saginata* stefins showed 95.9% and 79.6% homology, respectively. The C-terminal sequence of *T*. *asiatica* stefin2 revealed 82.1% and 98.9% homology with the *T*. *solium* stefin and *T*. *saginata* stefin, respectively. Large gene families often include both secreted and non-secreted members [42]. It was proposed that *T*. *asiatica* stefin2 with an N-terminal extension was a secreted protein. It has been reported that *Fasciola gigantica* stefin can be secreted [17, 18]. The present study does not provide evidence that *T*. *asiatica* stefin2 is a secreted protein and it remains to be investigated in future experiments.

Analysis of the amino acid sequences showed that platyhelminth and nematode cystatins had a signal peptide, except for *S*. *japonicum* cystatin1 (Fig 2A). Like chicken cystatin, whose crystallographic structure was the first to be resolved [32], platyhelminth cystatins had three conserved catalytic sites (G, QxVxG, and PW) in the domain that bind and inhibit proteases of the papain family (Fig 2A). The important role of conserved PW residues as part of the proteinase-binding site in the second hairpin loop has been confirmed previously [43, 44]. However, a Gly (G) occurred in place of Trp (W) in *S*. *japonicum* cystatin1, whereas a D residue occurred in place of a P residue in *H*. *microstoma* cystatin. More interestingly, PW residues were absent in *S*. *mediterranea* cystatin1 and *S*. *mansoni* cystatin1 (Fig 2A).

Notably, only one member of the cystatin superfamily, cystatin, was identified in *H*. *microstoma* and its key activity site had changed from PW residues to DW residues (Fig 2A), suggesting lack of catalytic activity. This raised a question of how *H*. *microstoma* inhibits various cysteine proteases. Previous studies have shown that stefins are primarily intracellular, while cystatins are found primarily in body fluids [40]. In plants, most cystatins gain function as inhibitors of both endogenous and exogenous cysteine proteases [40]. Additionally, there are numerous examples of loss of one or both lineages of cystatins and stefins, for example in *Apicomplexa* and *Fungi* [40]. A number of these pathogens have horizontally acquired bacterial chagasins for regulation of proteolysis [45]. It is speculated that the *H*. *microstoma* may inhibit various proteases either through its cystatin, which gains function as an inhibitor of both endogenous and exogenous cysteine proteases, like some plant cystatins, or horizontally acquiring the host’s cystatin superfamily, as in fungi [40].

Three different spliced cystatin proteins were deduced from electronic annotation in *S*. *mansoni* (S2 Fig). However, only *S*. *mansoni* cystatin2 contained all the characteristics of type 2 (cystatin-like doman, signal peptide and one or two conserved disulfide bonds) (Fig 2A). Similarly, *S*. *mediterranea* cystatin2 had all the characteristics of cystatins, but cystatin1 was lacking a PW activity site (Fig 2A). These results suggest that cystatins may have a functional divergence in *S*. *mansoni* and *S*. *mediterranea*. However, their functions have not been elucidated.

It was interesting to note that the size of cystatins in cestodes ranged from 274 to 295 amino acids (Table 2), substantially larger than those found in other species with 120 amino acid residues. Cestode cystatins exhibited a long sequence at the 3’ end after the conserved PW residues motif (Fig 2B, also see S2 Fig for a complete list). Searching for similarities within the amino acid sequences of each cestode cystatin revealed the presence of two tandem repeated domains (Fig 2B). However, the second tandem repeated domain did not contain a cystatin-like domain (G, QXVXG, PW). The mammalian kininogens are molecules with a high molecular weight containing multiple cystatin-type domains, which belong to members of family 3 of the cystatin superfamily [46]. Cystatin with multiple cystatin-like domains has also been reported in *F*. *hepatica* [47]. Cestode cystatins with one conserved domain did not belong to multi-domain cystatins, although they had two tandem repeated domains. The previously proposed evolutionary model hypothesized that the multi-domain cystatins originated from their stepwise evolution [48]. However, a new model of the evolution of the multi-domain cystatins considered domain duplication as a major mechanism for their origin [40]. The second tandem repeated domain throughout cestode cystatins may have originated from the cystatin domain, in the same manner as multi-domain cystatins.

There are two or three cystatin-like proteins in each planarian, nematode and trematode species (Table 2). However, there was only one cystatin protein with two tandem repeated domains in each cestode species. The results suggest that the repeated domain may have special functions. Additionally, mammals have many more copies of cystatin genes than do platyhelminths. For example, humans contain 17 members, suggesting gene expansions of the cystatin superfamily.

The other typical characteristic of cystatins is that they possess one or two conserved disulfide bridges. The analysis of cystatins in vertebrates, trematodes, nematodes, and planarians demonstrated that they had one or two conserved disulfide bonds (Fig 2A). However, the putative disulfide bonds in cestode cystatins were not conserved (Fig 2A, also see S1 Table for a complete list), like basal metazoans and plant cystatins [40]. This result indicates that structure of cestode cystatins is quite complex and is not consistent with previous evolutionary studies, which proposed that the disulfide bridges have been conserved in all cystatins [41]. It is well known that disulfide bonds have roles in the structure, stability and biological function of some proteins. Therefore, it is reasonable to believe that the putative disulfide bonds in cestode cystatins (S1 Table) may be related to their structure, stability and biological function, although they were not conserved.

Above results of comparison of amino acid sequences of cystatin superfamily revealed that the cystatin sequences were poorly conserved in platyhelminth species, except for the conserved cystatin-like domain (S2 Fig). However, the members of stefins were better conserved among these species (S1 Fig), a finding which was consistent with previous reporting [40].

### Phylogenetic analysis of platyhelminth cystatin superfamily

**Fig 3:**
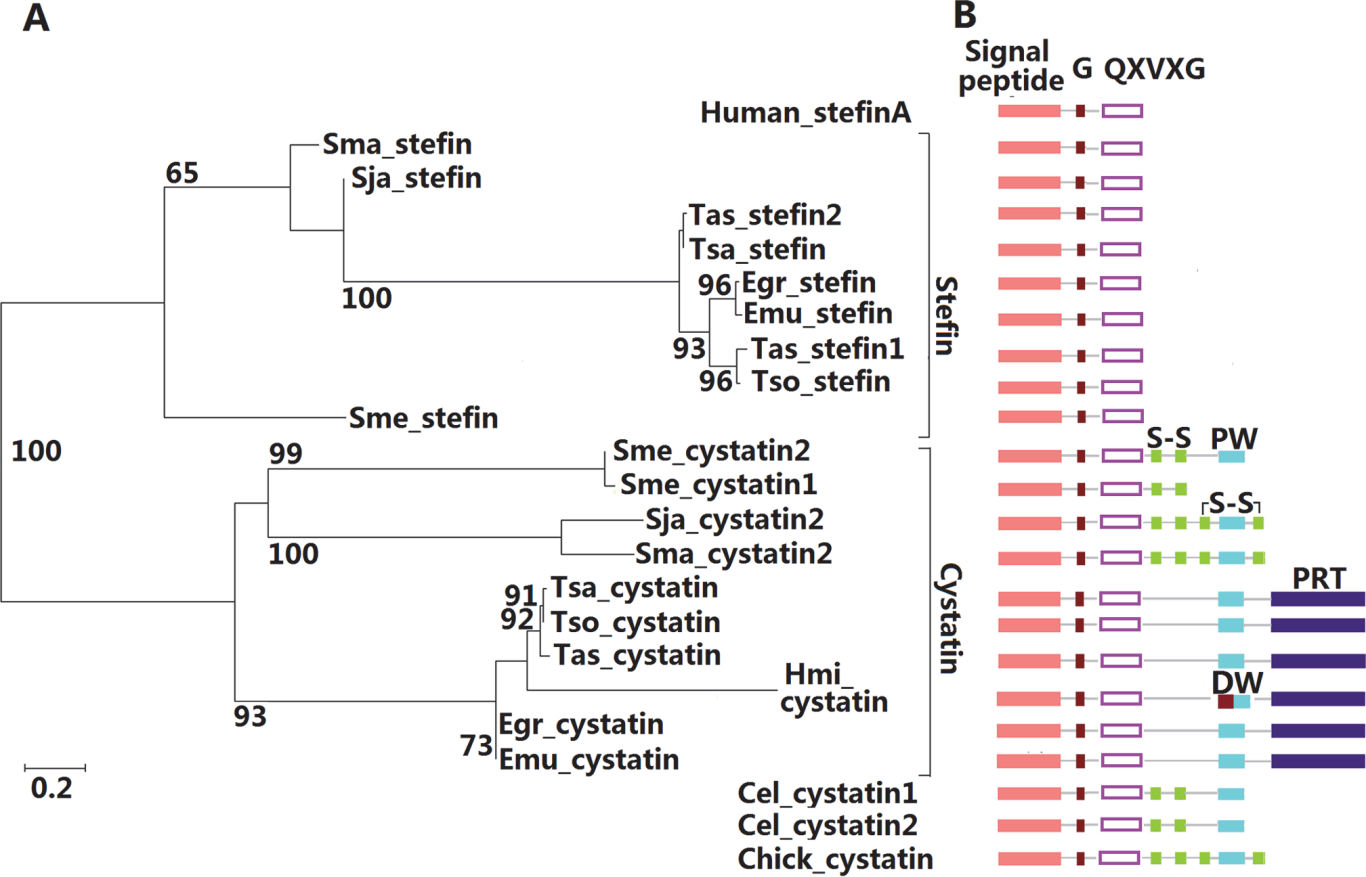
Phylogenetic analysis (A) and architecture (B) of platyhelminth cystatin superfamily. (A) Phylogenetic tree derived from the alignment of complete predicted proteins sequences. Nodes with confidence values greater than 60% are indicated based on maximum likelihood (ML). Subfamilies are indicated at the right. (B) The fragment from Gly (G) to Cys (C) or Pro-Trp (PW) residue represent predicted cystatin-like domain. The RPT represent tandem repeated domains. Disulfide bonds between conserved cysteines are denoted. The typical motifs for Stefin (human stefin A) and cystatin (chicken cystatin) subfamily members are represented on top and below, respectively. The first three letters: (Egr) *E*. *granulosus*; (Emu) *E*. *multilocularis*; (Sja) *S*. *japonicum*; (Sma) *S*. *mansoni*; (Sme) *S*. *mediterranea*; (Tas) *T*. *asiatica*; (Tsa) *T*. *saginata*; (Tso) *T*. *solium*; (Hmi) *H*. *microstoma*; (Cel) *C*. *Elegans*.

The phylogenetic tree of the platyhelminth cystatin superfamily was constructed and is shown in Fig 3A. Although *S*. *japonicum* cystatin1 was annotated to have a cystatin-like domain, no signal peptide and cystatin-like domain was predicted using SMART. Thus, it was discarded when the tree was constructed. For *S*. *mansoni*, one of three splicing forms of cystatin, with an intact cystatin-like domain was chosen to construct the tree. The phylogenetic analysis clearly showed that the members of platyhelminth cystatin superfamily were divided into two independent groups, the stefin and cystatin subfamilies. The stefins in trematodes, nematodes and cestodes branched together in a clade discrete from the planarian one. All platyhelminth species had a single copy for the stefin except for *T*. *asiatica* with two stefins (stefin1 and stefin2). All stefin genes in *T*. *solium*, *T*. *saginata* and *T*. *asiatica* (cestodes) were confirmed by PCR using their respective cDNA as template (Fig 1). The results showed that two stefin genes, stefin 1 and stefin 2, were really expressed in *T*. *asiatica* (Table 2) and provided evidence to rule out the possibility of incorrect genome assembly. The phylogenetic analysis showed that *T*. *asiatica* stefin1 was closely related to *T*. *solium* stefin, and *T*. *asiatica* stefin2 to *T*. *saginata* stefin. The results of the genomic organization and the phylogenetic analysis supported the idea that *T*. *asiatica* stefin2 may have arisen during evolution before the separation of these cestode parasites.

Phylogenetic analysis revealed that *Schistosoma* and planarian cystatins belong to the same group and a putative gene duplication event may have occurred before or at the common ancestor of the planarian. It was noteworthy that a single copy of cystatin in each cestode species with two tandem repeated domains but no conserved disulfide bonds constituted an independent clade (Fig 3A and 3B). The diverse groups of proteins that are homologous to chicken cystatins have had their evolution schemes constructed in previous studies [40, 41]. However, the cystatin structures with two tandem repeated domains and without conserved disulfide bonds, like the cestode cystatins described in this study, have not been previously reported. The results presented here imply that the cestode cystatins may be considered a new subfamily.

### Tertiary structure of *T*. *solium* cystatin showed some differences with chicken cystatin

**Fig 4:**
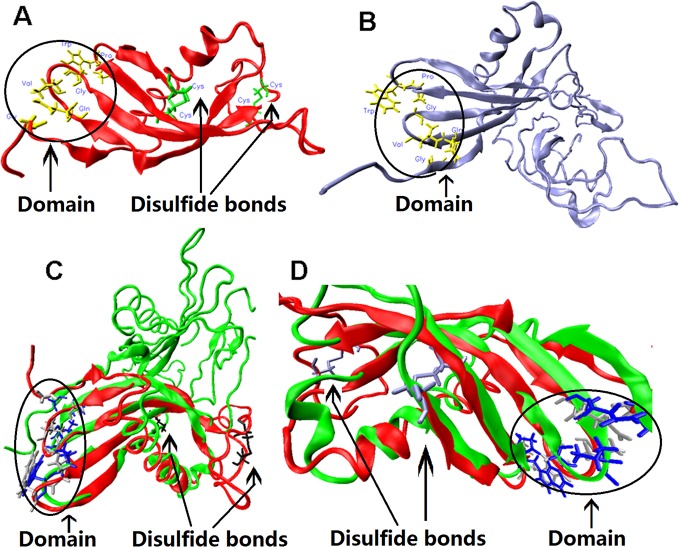
Comparison of tertiary structures for chicken and *T*. *solium* cystatins. The 3D structures for chicken cystain (A) and *T*. *solium* cystatin (B) are shown. The cystatin models of chicken and *T*. *solium* are superimposed in (C). The opposite side of the (C) map are enlarged and shown in (D).

Cestode cystatins with two tandem repeated domains and without conserved disulfide bonds have not been reported previously. It is intriguing to explore whether they have similar structural features to chicken cystatin. The crystal structure of chicken cystatin consists mainly of a five string α-helix and a five-stranded β-pleated sheet [1]. N-terminal Gly residue, QXVXG residues sited at the first β-hairpin loop and Pro-Trp residues sited at the second β-hairpin loop forming a hydrophobic edge to penetrate into the active site cleft of papins have been discussed in detail [43, 44, 49, 50]. The 3D structure of chicken cystatin [32] and *T*. *solium* cystatin are shown in Fig 4A and 4B, respectively. Although the structure of the extra C-terminal repeat domain of *T*. *solium* cystatin was difficult to predict, a superimposed diagram of 3D models for *T*. *solium* cystatin and chicken cystatin indicates that their papain inhibitory loop (G, QXVXG, PW) almost overlaps (Fig 4C). Although no conserved disulfide bond was found in *T*.*solium* cystatin, the models of *T*.*solium* cystatin and chicken cystatin corresponded at the site of the first disulfide bridge of the chicken cystatin, which may be explained by other kinds of bonds, such as a hydrogen bond. The two models were different regarding the location of the second disulfide bridge of chicken cystatin (Fig 4D). The same results were also obtained for other cestode cystatins (S3 Fig). These results showed that the models of cestode cystatins and chicken cystatin had both similarities and differences, suggesting that the biochemistry of cestode cystatins could be similar to chicken cystatin in its inhibitory function and they may have additional functional roles. This result is in disagreement with a recent study that has suggested it was not possible to identify cystatin homologs in *E*. *granulosus*, *H*. *microstoma* and *T*. *solium* [19]. In addition, excepting cestode cystatins, all predicted models of platyhelminth stefins and cystatins exhibited the similar conserved α-helix and β-pleat and the functional hydrophobic edge features that find in the human stefin [33, 51] and chicken cystatin models [32], respectively (not shown). Previous studies have shown that the structure of the plant inhibitor oryzacystatin possesses the same cystatin fold as animal cystatin, which has the ability to inhibit cysteine proteinase [52]. These results suggest that the biochemistry of the platyhelminth cystatin superfamily could be similar to chicken cystatin and human stefin in its inhibitory function, and it is possible that cestode cystatins may have further, as yet undefined, functional roles.

In this study, the cystatin superfamily was identified in platyhelminths using extensive genomic data, and divided into stefin and cystatin subfamilies. It had many important characteristics in common with the mammalian cystatin superfamily, except for cestode cystatins, which had two tandem repeated domains, but lacked the conserved disulfide bond. The specific characteristic of cestode cystatins was also indicated in phylogenetic analysis where they formed a separate clade. Additionally, the tertiary structure of *T*. *solium* cystatin showed both similarities and differences when compared to chicken cystatin. This study provided the first insights into the abundance, gene structure, 3D model structure, evolution and functional diversification of the cestode cystatin superfamily. The biochemistry of platyhelminth stefins and cystatins needs to be further characterized, especially for the unusual cestode superfamily.

## Supporting Information

S1 Fig Alignment of platyhelminth stefin superfamily. (TIF)

S2 Fig Alignment of platyhelminth cystatin superfamily. (TIF)

S3 Fig Superimposing 3D models of chicken and cestode cystatins. The 3D structures for chicken cystain are shown in yellow, cestode cystains in other colors. Conserved cysteines (in green stick view) are displayed. The three parts of the papain binding domain (in stick view) are surrounded by ellipses.
(TIF)

S1 Table Putative the disulfide bonds in platyhelminth cystatins. (DOC)
