# Supplementary figures and images for: Comparative Analysis of Cystatin Superfamily in Platyhelminths
Source: PLoS One. 2015 Apr 8;10(4):e0124683. doi: 10.1371/journal.pone.0124683 (PMC4390278; doi:10.1371/journal.pone.0124683)

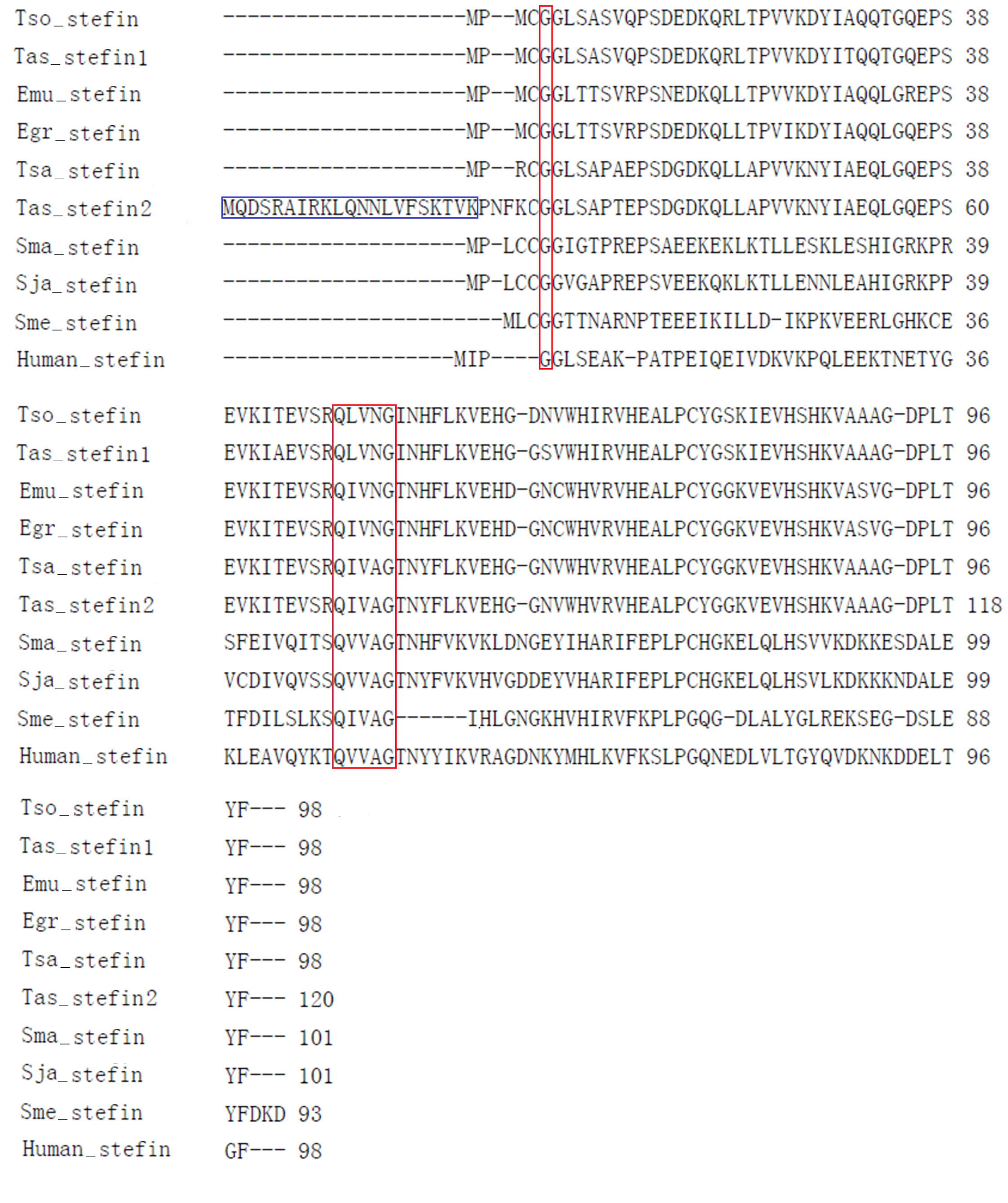

Supplement: S1 Fig — (TIF) [file pone.0124683.s001.tif]

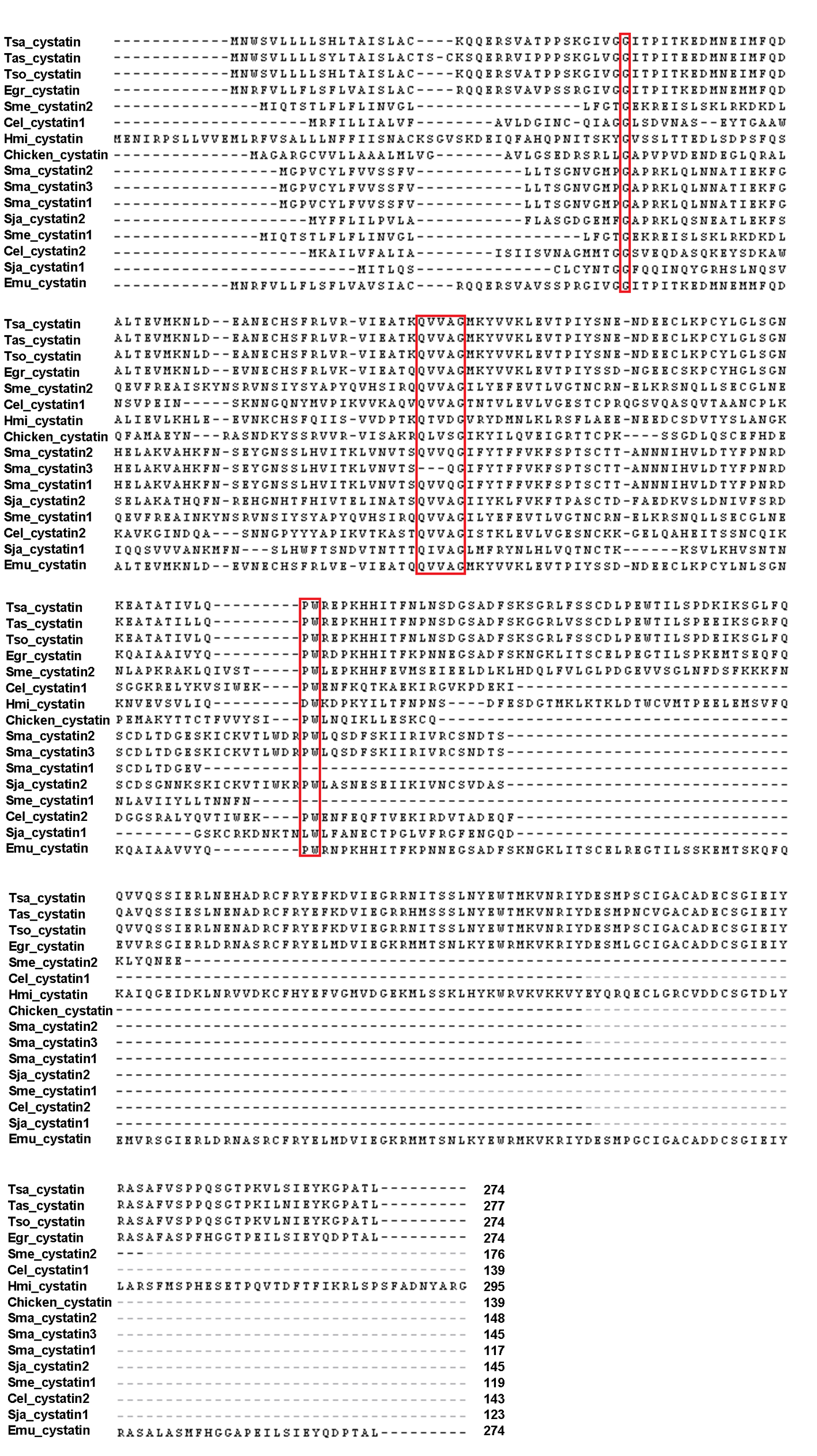

Supplement: S2 Fig — (TIF) [file pone.0124683.s002.tif]

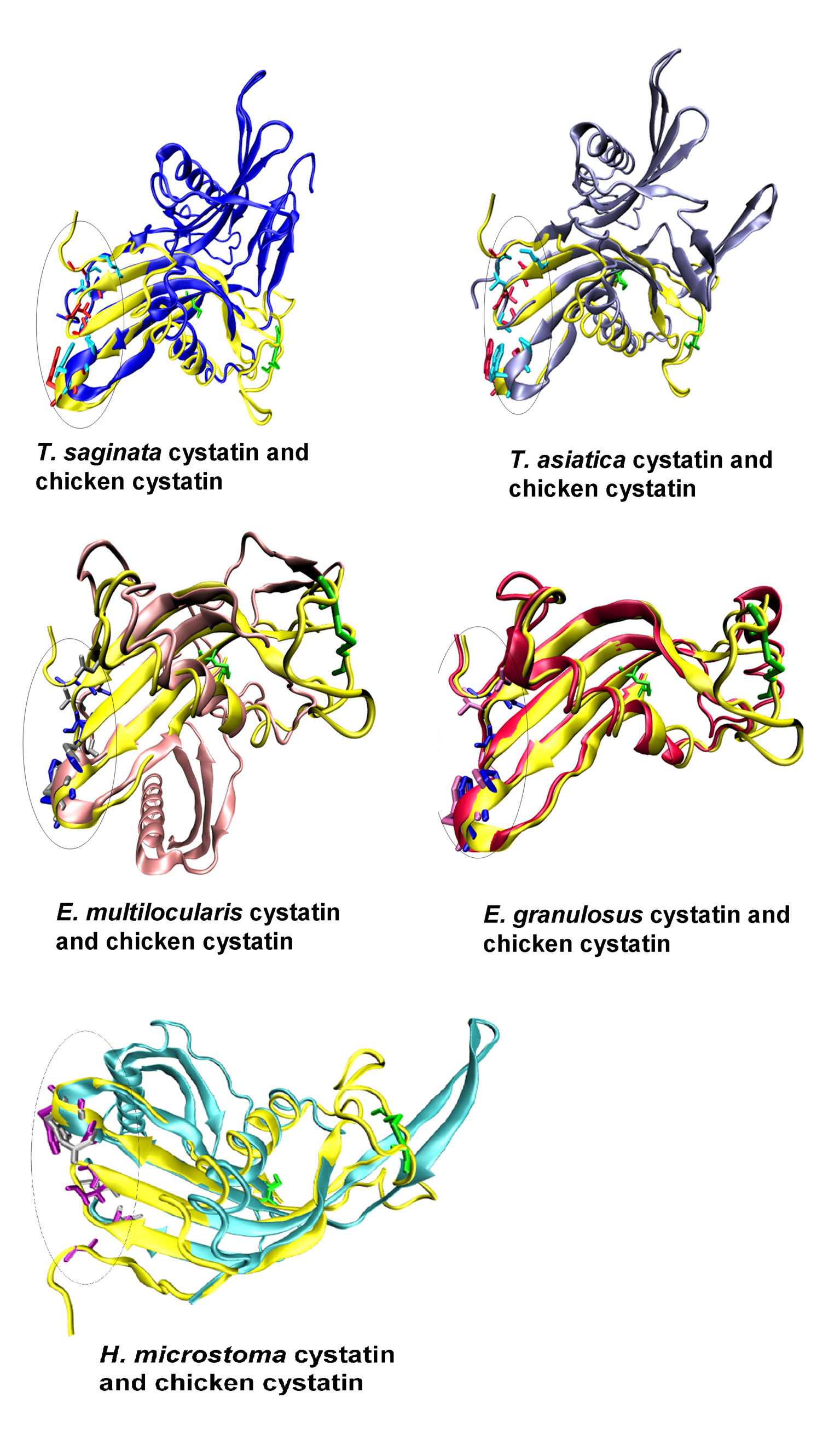

Supplement: S3 Fig — The 3D structures for chicken cystain are shown in yellow, cestode cystains in other colors. Conserved cysteines (in green stick view) are displayed. The three parts of the papain binding domain (in stick view) are surrounded by ellipses. (TIF) [file pone.0124683.s003.tif]
