# Supplementary material for: Comparative Analysis of Cystatin Superfamily in Platyhelminths
Source: PLoS One. 2015 Apr 8;10(4):e0124683. doi: 10.1371/journal.pone.0124683 (PMC4390278; doi:10.1371/journal.pone.0124683)
Supplement: S1 Table — (DOC) [file pone.0124683.s004.doc]

**S1 Table. Putative the disulfide bonds in platyhelminth cystatins**

| Cystatin | Location | Sequence | Located at conserved site or not |
| --- | --- | --- | --- |
| Tso_cystatin | 19 - 233 | AISLACKQQER - ESMPSCIGACA | No |
|  | 68 - 159 | DEANECHSFRL - RLFSSCDLPEW | No |
|  | 107 - 241 | ENDEECLKPCY - ACADECSGIEI | No |
|  | 111 - 237 | ECLKPCYLGLS - SCIGACADECS | No |
| Tsa_cystatin | 19 - 111 | AISLACKQQER - ECLKPCYLGLS | No |
|  | 68 - 159 | DEANECHSFRL - RLFSSCDLPEW | No |
|  | 107 - 241 | ENDEECLKPCY - ACADECSGIEI | No |
|  | 195 - 237 | EHADRCFRYEF - SCIGACADECS | No |
| Tas_cystatin | 19 - 114 | AISLACTSCKS - ECLKPCYLGLS | No |
|  | 22 - 240 | LACTSCKSQER - NCVGACADECS | No |
|  | 71 - 162 | DEANECHSFRL - RLVSSCDLPEW | No |
|  | 110 - 244 | ENDEECLKPCY - ACADECSGIEI | No |
|  | 198 - 236 | ENADRCFRYEF - ESMPNCVGACA | No |
| Emu_cystatin | 19 - 107 | AVSIACRQQER - DNDEECLKPCY | No |
|  | 111 - 241 | ECLKPCYLNLS - ACADDCSGIEI | No |
|  | 159 - 233 | KLITSCELREG - ESMPGCIGACA | No |
|  | 195 - 237 | RNASRCFRYEL - GCIGACADDCS | No |
| Egr_cystatin | 19 - 241 | AISLACRQQER - ACADDCSGIEI | No |
|  | 107 - 111 | DNGEECSKPCY - ECSKPCYHGLS | No |
|  | 159 - 195 | KLITSCELPEG - RNASRCFRYEL | No |
|  | 233 - 237 | ESMLGCIGACA - GCIGACADDCS | No |
| Hmic_cystatin | 31 - 253 | IISNACKSGVS - RCVDDCSGTDL | No |
|  | 84 - 245 | EEVNKCHSFQI - YQRQECLGRCV | No |
|  | 123 - 207 | ENEEDCSDVTY - RVVDKCFHYEF | No |
|  | 177 - 249 | KLDTWCVMTPE - ECLGRCVDDCS | No |
| Smed_cystatin 1 | 87 - 101 | LVGTNCRNELK - QLLSECGLNEN | Yes |
| Smed_cystatin 2 | 87 - 101 | LVGTNCRNELK - QLLSECGLNEN | Yes |
| Sjap_cystatin 1 | 77 - 109 | LVQTNCTKKSV - LFANECTPGLV | Yes |
| Sjap_cystatin 2 | 87 - 107 | FTPASCTDFAE - FSRDSCDSGNN | Yes |
|  | 117 - 140 | NKSKICKVTIW - IKIVNCSVDAS | Yes |
| Sman_cystatin 1 | 90-110 | FSPTSCTTANN - PNRDSCDLTDG | Yes |
| Sman_cystatin 2 | 90 - 110 | FSPTSCTTANN - PNRDSCDLTDG | Yes |
|  | 120 - 143 | GESKICKVTLW - IRIVRCSNDTS | Yes |
| Sman_cystatin 3 | 87 - 107 | FSPTSCTTANN - PNRDSCDLTDG | Yes |
|  | 117 - 140 | GESKICKVTLW - IRIVRCSNDTS | Yes |
| Chicken_cystatin | 94 - 104 | IGRTTCPKSSG - GDLQSCEFHDE | Yes |
|  | 118 - 138 | AKYTTCTFVVY - LLESKCQ | Yes |
